# Evaluation of Knowledge and Attitudes of the Population of Tabuk City Regarding Parkinson’s Disease: A Cross-Sectional Study

**DOI:** 10.7759/cureus.46442

**Published:** 2023-10-03

**Authors:** Muhammad Moosa M Qasim, Abdulrahman A Daghreeri, Omayrah A Alanazi, Lubna I AlOmari, Khalid F Alzibali, Wafa A Alrezqi, Raghad Faraih Albalawi, Khadijah M AlHadi, Yahya Alhasan Alhazmi, Hayat M Refai

**Affiliations:** 1 Medicine, King Salman Armed Forces Hospital, Tabuk, SAU; 2 Medicine, Jazan University, Jazan, SAU; 3 Medicine, University of Tabuk, Tabuk, SAU; 4 Medicine, King Abdulaziz University, Jeddah, SAU; 5 Medicine, Albaha University, Albaha, SAU; 6 Pediatrics, Faculty of Medicine, University of Tabuk, Tabuk, SAU; 7 Medicine, Umm Al-Qura University, Al Qunfudhah, SAU

**Keywords:** public, saudi arabia, awareness, knowledge, parkinson’s disease

## Abstract

Background: The lack of awareness and information about PD may be a barrier to early diagnosis and the delivery of the best care to patients with the condition, given its rising prevalence. In order to determine the variables that are connected to these parameters, this study sought to ascertain the general public's knowledge and awareness of PD in Tabuk City.

Methods: In Tabuk City, a cross-sectional demographic survey was carried out. A validated structured questionnaire was used to interview adult respondents by random sampling regarding specific knowledge, attitudes, and awareness related to Parkinson's disease. According to the density of the city, a total of 426 members of the general population were chosen at random and interviewed by skilled interviewers.

Results: Age and educational attainment were independently linked to PD awareness. Bachelor's degree subjects and those between the ages of 18 and 45 displayed a greater awareness of PD. Those above 60 and those between the ages of 46 and 60 lacked sufficient knowledge. The majority of participants demonstrated adequate understanding and awareness of PD in their respective occupations.

Conclusions: Age, gender, occupation, and level of education were all adequately covered by knowledge and understanding of PD. To increase public knowledge, attitudes, and awareness of PD, however, suitable educational tactics and approaches targeting particular subgroups are required.

## Introduction

Parkinson's disease (PD) is an intricate, progressive neurological disorder characterized by tremors, stiffness, and bradykinesia. Some sufferers may develop postural instability as the illness gets worse. Our understanding of PD has progressively expanded. James Parkinson first described it in 1817, and Jean-Martin Charcot later provided further elaboration [1].

The condition has a serious clinical effect on individuals, their caregivers, and their families because of its increasing degenerative consequences on muscular control and mobility. Although the existence of nonmotor symptoms implies neuronal degeneration in nondopaminergic areas, it is thought that PD's motor symptoms are caused by the lack of striatal dopaminergic neurons. As a symptom complex, parkinsonism is a phrase that describes the motor symptoms of PD, such as resting tremor, cogwheeling, and bradykinesia. The primary cause of parkinsonism is PD, although there are a variety of secondary causes as well, including diseases that mirror PD and drug-induced causes [2].

Estimates of the annual incidence of PD vary, with reported rates ranging from five to over 35 new cases per 100,000 individuals. Between the sixth and ninth decades of life, the incidence increases by five to 10 times. The likelihood of PD increases with age. A meta-analysis involving four North American groups found that the prevalence rose from under 1% of men and women aged 45-54 to 4% of men and 2% of women aged 85 and higher. Mortality rates in individuals with PD do not significantly rise during the first ten years following diagnosis compared to unaffected individuals, but they increase thereafter. The prevalence of PD is expected to more than double over the next 20 years as the world's population ages. The societal and financial cost of PD will increase along with it if new therapies, treatments, or prevention measures are not discovered [3,4].

Dopaminergic neurons are pathologically lost in PD, primarily within the substantia nigra pars compacta (SNpc), a midbrain region that also hosts Lewy bodies-cytoplasmic inclusions containing insoluble alpha-synuclein clumps. However, more severe disease in other regions of the brain, such as non-dopaminergic neurons, distinguishes PD from other forms of the condition. Although non-motor symptoms like smell blindness, functional constipation, major depressive disorder, and REM sleep disorder can manifest years before motor deficiencies, the clinical diagnosis of PD is primarily based on motor features like a slowly progressing resting tremor, cogwheeling, and bradykinesia. Other non-motor symptoms, such as pain, autonomic dysfunction, and cognitive impairment, may also appear in the late stage of the illness [3,5].

There are very few studies on the understanding of PD among caretakers. These studies revealed that the level of PD knowledge among the caretakers was incredibly low [6,7]. The majority of carers claim that, now that their loved ones have been diagnosed, they don't have adequate knowledge. Lack of general information about PD patients and their caretakers may cause communication problems between the doctor, patient, and carer. This situation directly harms both the patient's daily activities and the disease therapy [8].

The PD diagnostic delays and treatment discrepancies that are now unexplained may be greatly influenced by knowledge and attitudes about the disease. An Alzheimer's disease study found that increased help-seeking activity was associated with higher degrees of condition awareness [9]. Few people are aware of PD, and many associate it with stigma, according to past studies on knowledge and attitudes in the community [10]. These could act as further barriers to care, particularly for underprivileged minorities. If diagnostic delays and treatment disparities are lessened, minorities with PD may experience a higher quality of life. A deeper understanding of the care barriers will be necessary to develop solutions that will achieve those goals. Cultural competency training sessions, a vital part of healthcare professional education, can be improved by healthcare educators by learning about various racial and ethnic groups' attitudes toward health [11].

Literature on knowledge and attitude in the assessment of PD in Saudi Arabia is scarce. Hence, the objective of this study is to bridge the knowledge gap and enhance comprehension of this subject.

## Materials and methods

Study design and setting

The cross-sectional study was used to evaluate the level of PD knowledge and awareness among the general population in Tabuk City, KSA. Data were collected at public areas chosen randomly between June 1 and August 30, 2023.

Sample size technique

The sample size for this study was determined using the following formula:

n = (Z^2^ * p * (1-p))/E^2^

Where: n = sample size Z = Z-score for a 95% confidence level (1.96) p = estimated prevalence of knowledge and attitude towards the effects of alcohol in the population (50%) E = margin of error (5%) Based on the above formula, the minimum sample size required for this study is 384 participants. However, for a 95% confidence level with a 5% margin of error, we aimed to include 426 participants in our study.

Eligibility criteria

This study included all persons (18 years of age and older) living in Tabuk City who were willing to participate in the study. Individuals who are unable to offer informed consent and Individuals who are unable to communicate effectively in Arabic or English were excluded.

Data collection tools and techniques

A random sampling technique was employed in this study. Participants were selected randomly from the general public of Tabuk City. Those who met the inclusion criteria were invited to participate in the study. The study variables included sociodemographic variables such as age, gender, education level, and nationality, as well as knowledge-related variables such as familiarity with PD, signs and symptoms of PD, risk factors, and treatment options.

The structured interview questionnaire used in this study was developed based on a review of prior research and expert opinions, as detailed in the questionnaire provided in the appendix. The questionnaire was pre-tested on 30 participants of the target population that were not included into study to ensure its clarity, relevance, and validity (piloting study). The result of Cronbach's alpha was 0.76 which was an acceptable level.

Data analysis plan

The data were analyzed using SPSS version 28 (IBM Corp., Armonk, NY). Descriptive statistics such as frequencies and percentages were used to summarize the sociodemographic and knowledge-related variables. Inferential statistics such as the Chi-square test and logistic regression analysis were used to identify any associations between sociodemographic variables and knowledge level. If the p-value was less than 0.05, all statistical test findings were deemed significant.

Ethical consideration

This study obtained ethical approval from the Institutional Review Board (IRB) of King Salman Armed Forces Hospital, as indicated by approval number KSAFH-REC-2023-514. Before participating in the trial, all study participants provided oral informed consent. The questionnaire was collected from respondents and treated with confidentiality while keeping optimal privacy.

## Results

A total of 426 responses were received. Table 1 shows the sociodemographic characteristics of the studied group. The age ranged from 18 to above 60 years old; the females represented n=264 (62%), while the males were n=162 (38%).

**Table 1:** Sociodemographic characteristics of the study participants (n = 426) N: Numbers of Participants; %: Percentages

Variables	Categories	N	%
Gender	Female	264	62
	Male	162	38
Age groups (years)	18-30	328	77
	31-45	65	15.3
	46-60	30	7
	>60	3	0.7
Marital Status	Single	307	72.1
	Married	94	22.1
	Divorced	18	4.2
	Widowed	7	1.6
Education Level	Primary education	10	2.3
	Secondary education	133	31.2
	Master’s degree	12	2.8
	Bachelor's degree	269	63.2
	Doctoral degree	2	0.5
Occupation	Student	246	57.7
	Unemployed	62	14.6
	Employed	104	24.4
	Retired	14	3.3

As illustrated in Table 2, the participants showed good Knowledge and Awareness about PD. The majority of the participants heard about PD (n=356, 83.6%), and 93.66% (n=399) of the participants showed that the common symptom of PD is tremors, however 54.7% (n=233) of the participants thought that PD could be cured, and about 56.57% (n=241) of them see that exercise is not a risk factor for developing PD, the majority of the participants saw that PD affect a person's cognitive abilities (n=254, 59.6%); moreover, 40.1% (n=171) of the participants saw that PD more common in men than women, while 48.15% (n=205) saw that it is common in both.

**Table 2:** Knowledge and Awareness about Parkinson's disease N: Numbers of Participant; %: Percentages

Question	Response	N	%
Have you ever heard of Parkinson's disease?	Yes	356	83.6
	No	70	16.4
Do you know what Parkinson's disease is?	Yes	259	60.8
	No	167	39.2
Which of the following is a common symptom of Parkinson's disease?	Hearing loss	61	14.32
	Stomach pain	29	6.81
	Tremors	399	93.66
	Vision problems	102	23.94
Can Parkinson's disease be cured?	Yes	233	54.7
	No	193	45.3
Which of the following is not a risk factor for developing Parkinson's disease?	Age	142	33.33
	Exercise	241	56.57
	Family history	101	23.71
	Smoking	119	27.93
Which of the following is a treatment for Parkinson's disease?	Antibiotics	119	27.93
	Chemotherapy	68	15.96
	Surgery	86	20.19
	Medication	349	81.92
Can Parkinson's disease affect a person's cognitive abilities?	Yes	254	59.6
	No	172	40.4
Is Parkinson's disease more common in men or women?	Men	171	40.1
	Women	50	11.7
	Both	205	48.1
What causes Parkinson's disease?	Exposure to toxins	71	16.67
	Genetics	155	36.38
	Poor diet	70	16.43
	All of the above	257	60.33
Have you or any of your family members ever been diagnosed with Parkinson's disease?	Yes	39	9.2
	No	387	90.8

From Table 3, we noticed that the internet represented the most common source of knowledge about PD, followed by healthcare professionals.

**Table 3:** Sources of Information about Parkinson's disease N: Numbers of Participant; %: Percentages

Variables	Response	N	%
TV/Radio:	Never	126	29.6
	Often	62	14.6
	Rarely	130	30.5
	Sometimes	108	25.4
Internet:	Never	48	11.3
	Often	204	47.9
	Rarely	62	14.6
	Sometimes	112	26.3
Books/Magazines:	Never	110	25.8
	Often	72	16.9
	Rarely	148	34.7
	Sometimes	96	22.6
Healthcare professionals (e.g., doctors, nurses):	Never	78	18.3
	Often	114	26.8
	Rarely	84	19.7
	Sometimes	150	35.2
Friends/Family:	Never	90	21.1
	Often	94	22.1
	Rarely	80	18.8
	Sometimes	162	38.0

In Table 4, we noticed that the answer about PD is a result of poor lifestyle choices was not clear where 31.7% (n=135) agreed, while 36.4% (n=155) disagreed about this sentence, moreover 71.1% (n=303) of the participants saw that people with PD should be supported and helped by the community, the majority of the participants agreed that people with PD can lead fulfilling lives with proper treatment and support (n=306, 71.9%), about people with PD should not be stigmatized or discriminated against 67.1% (n=186) of the participants showed agreement, and 69.5% (n=296) of the participants showed willing to support and help people with PD.

**Table 4:** Attitude of participants towards Parkinson’s disease N: Numbers of Participant; %: Percentages

Variables	Response	N	%
Parkinson's disease is a result of poor lifestyle choices.	Strongly agree	28	6.6
	Neutral	136	31.9
	Agree	107	25.1
	Strongly disagree	71	16.7
	Disagree	84	19.7
People with Parkinson's disease should be supported and helped by the community.	Strongly agree	162	38.0
	Neutral	64	15.0
	Agree	141	33.1
	Strongly disagree	37	8.7
	Disagree	22	5.2
People with Parkinson's disease can lead fulfilling lives with proper treatment and support.	Strongly agree	124	29.1
	Neutral	50	11.7
	Agree	182	42.8
	Strongly disagree	24	5.6
	Disagree	46	10.8
More education and awareness should be provided to the public about Parkinson's disease.	Strongly agree	158	37.1
	Neutral	48	11.3
	Agree	152	35.7
	Strongly disagree	39	9.2
	Disagree	29	6.7
People with Parkinson's disease should be encouraged to participate in social activities and events.	Strongly agree	141	33.1
	Neutral	63	14.8
	Agree	155	36.3
	Strongly disagree	31	7.3
	Disagree	36	8.5
People with Parkinson's disease should be able to work and be productive members of society.	Strongly agree	113	26.5
	Neutral	93	21.8
	Agree	164	38.5
	Strongly disagree	25	5.9
	Disagree	31	7.3
People with Parkinson's disease should not be stigmatized or discriminated against.	Strongly agree	146	34.3
	Neutral	74	17.4
	Agree	140	32.8
	Strongly disagree	41	9.6
	Disagree	25	5.9
I am willing to support and help people with Parkinson's disease	Strongly agree	141	33.1
	Neutral	57	13.4
	Agree	155	36.4
	Strongly disagree	41	9.6
	Disagree	32	7.5

We can see from Table 5 that the female participants had much more information than the male participants concerning PD (p = 0.033), its most prevalent symptoms (p = 0.001), and whether it can be cured. Is PD more prevalent in men or women? (p = 0.005), the risk factor for developing the condition (p = 0.003), and so on (p = 0.015). And have you or any members of your family ever received a PD diagnosis? (p = 0.026). Do you know what PD is? (p=0.207) revealed a non-significant association between genders, but which of the following is a treatment for PD? did not (p = 0.265). Can a person's cognitive abilities be impacted by PD? What causes PD? (p = 0.276) and (p = 0.213).

**Table 5:** Association between the participants knowledge and awareness according to gender **p<0.01 is statistically significant. Chi-square test was computed. *p<0.05 is statistically significant. Chi-square test was computed. F is Fisher’s exact test N: Numbers of Participant; %: Percentages

Variables	Responses Of the participants	Female (N=264, 62%)	Male (N=162, 38%)	P-value
Have you ever heard of Parkinson's disease?	Yes	228 (86.4%)	128 (79%)	0.033*
	No	36 (13.6%)	34 (21%)	
Do you know what Parkinson's disease is?	Yes	156 (59.1%)	103 (63.6%)	0.207 (F)
	No	108 (40.9%)	59 (36.4%)	
Which of the following is a common symptom of Parkinson's disease?	Hearing loss	43 (16.29%)	18 (11.11%)	0.001**
	Stomach pain	20 (7.585)	9 (5.56%)	
	Tremors	255 (96.59%)	144 (88.89%)	
	Vision problems	62 (23.48%)	40 (24.69%)	
Can Parkinson's disease be cured?	Yes	131 (49.6%)	133 (50.4%)	0.005 (F)
	No	102 (63%)	60 (37%)	
Which of the following is not a risk factor for developing Parkinson's disease?	Age	92 (34.85%)	50 (30.86%)	0.003**
	Exercise	162 (61.36%	79 (48.77%)	
	Family history	63 (23.86%)	38 (23.46%)	
	Smoking	68 (25.76%)	51 (31.48%)	
Which of the following is a treatment for Parkinson's disease?	Antibiotics	78 (29.55%)	52 (32.10%)	0.265
	Chemotherapy	44 (16.67%)	24 (14.81%)	
	Surgery	59 (22.35%)	42 (25.93%)	
	Medication	222 (84.09%)	127 (78.40%)	
Can Parkinson's disease affect a person's cognitive abilities?	Yes	153 (58%)	101 (62.3%)	0.213 (F)
	No	111 (42%)	61 (37.7%)	
Is Parkinson's disease more common in men or women?	Men	92 (34.8%)	79 (48.8%)	0.015*
	Women	32 (12.1%)	18 (11.1%)	
	Both	140 (53%)	65 (40.1%)	
What causes Parkinson's disease?	Exposure to toxins	43 (16.29%)	28 (17.28%)	0.276
	Genetics	78 (29.55%)	52 (32.10%)	
	Poor diet	39 (14.77%)	32 (19.75%)	
	All of the above	160 (60.61%)	97 (59.88%)	
Have you or any of your family members ever been diagnosed with Parkinson's disease?	Yes	18 (6.8%)	21 (13%)	0.026 (F)
	No	246 (93.2%)	141 (87%)	

As illustrated in Table 6, a significant association between the statements that PD is caused by poor lifestyle choices and that individuals with PD should be able to work and be productive members of society (p = 0.027 and p = 0.045, respectively) that was higher in females than in males. Regarding the replies to the other factors, there was no statistically significant difference between males and females (p > 0.05).

**Table 6:** Association between the participants attitude towards Parkinson’s disease according to gender *p<0.05 is statistically significant N: Numbers of Participants; %: Percentages

Variables	Responses Of the participants	Female (N=264, 62%)	Male (N=162, 38%)	P-value
Parkinson's disease is a result of poor lifestyle choices.	Strongly agree	10 (3.8%)	18 (11.1%)	0.027*
	Neutral	90 (34.1%)	46 (28.4%)	
	Agree	66 (25%)	41 (25.3%)	
	Strongly disagree	41 (15.5%)	30 (18.5%)	
	Disagree	57 (21.6%)	27 (16.7%)	
People with Parkinson's disease should be supported and helped by the community.	Strongly agree	98 (37.1%)	64 (39.5%)	0.145
	Neutral	38 (14.4%)	26 (16%)	
	Agree	98 (37.1%)	43 (26.5%)	
	Strongly disagree	19 (7.2%)	18 (11.1%)	
	Disagree	11 (4.2%)	11 (6.8%)	
People with Parkinson's disease can lead fulfilling lives with proper treatment and support.	Strongly agree	83 (41.4%)	41 (25.3%)	0.079
	Neutral	28 (10.6%)	22 (13.6%)	
	Agree	108 (40.9%)	74 (45.7%)	
	Strongly disagree	20 (7.6%)	4 (2.5%)	
	Disagree	25 (9.5%)	21 (13%)	
More education and awareness should be provided to the public about Parkinson's disease.	Strongly agree	106 (40.2%)	52 (32.1%)	0.114
	Neutral	22 (8.3%)	26 (16%)	
	Agree	96 (36.4%)	56 (34.6%)	
	Strongly disagree	23 (8.7%)	16 (9.9%)	
	Disagree	17 (6.4%)	12 (7.4%)	
People with Parkinson's disease should be encouraged to participate in social activities and events.	Strongly agree	92 (34.8%)	49 (30.2%)	0.063
	Neutral	31 (11.7%)	32 (19.8%)	
	Agree	104 (39.4%)	51 (31.5%)	
	Strongly disagree	19 (7.2%)	12(7.4%)	
	Disagree	18 (6.8%)	18 (11.1%)	
People with Parkinson's disease should be able to work and be productive members of society.	Strongly agree	74 (28%)	39 (24.1%)	0.045*
	Neutral	45 (17%)	48 (29.6%)	
	Agree	107 (40.5%)	57 (35.2%)	
	Strongly disagree	16 (6.1%)	9 (5.6%)	
	Disagree	22 (8.3%)	9 (5.6%)	
People with Parkinson's disease should not be stigmatized or discriminated against.	Strongly agree	91 (34.5%)	55 (34%)	0.341
	Neutral	39 (14.8%)	35 (21.6%)	
	Agree	88 (33.3%)	52 (32.1%)	
	Strongly disagree	28 (10.6%)	13 (8%)	
	Disagree	18 (6.8%)	7 (4.3%)	
I am willing to support and help people with Parkinson's disease	Strongly agree	97 (36.7%)	44 (27.2%)	0.187
	Neutral	31 (11.7%)	26 (16%)	
	Agree	95 (36%)	60 (37%)	
	Strongly disagree	25 (9.5%)	16 (9.9%)	
	Disagree	16 (6.1%)	16 (9.9%)	

## Discussion

Globally, PD is becoming more prevalent; hence, it is crucial to gauge how well-informed and aware the general public is of the condition. The disease affects older people more frequently, and its prevalence rises with age. Although the precise origin of PD is uncertain, a mix of hereditary and environmental factors is thought to be responsible [1]. We used 426 participants in our study to provide a previously validated questionnaire to the general population. Our research showed that participants generally exhibited strong knowledge and awareness of Parkinson's illness.

The majority of the participants heard about PD with a percentage of 83.6% (n=356), and 93.66% (n=399) of the participants showed that the common symptom of PD is tremors, our findings were supported by Park et al. [12], education levels showed a non-significant difference in their answers about knowledge and awareness of PD, these findings were supported by Youn et al. [13], however, 54.7% (n=233) of the participants thought that PD could be cured, these findings were in contrast with WHO [14], who stated that "There is no cure for Parkinson disease, but therapies including medicines, surgery, and rehabilitation can reduce symptoms", and about 56.57% (n=241) of them see that exercise is not a risk factor for developing PD, the majority of the participants saw that PD affect a person's cognitive abilities (n=254, 59.6%). These findings were on the same lines as those of Parajuli et al. [15], and 40.1% (n=171) of the participants saw that PD is more common in men than women. These results were supported by Miller and Cronin-Golomb, who stated that males are diagnosed with PD at a ratio of roughly 2:1, whereas 48.15% (n=205) stated that it is frequent in both men and women [16].

From our results, we noticed that the answer about whether PD is a result of poor lifestyle choices was not clear; 31.7% (n=135) agreed, while 36.4% (n=155) disagreed. Furthermore, 71.1% (n=303) of the participants saw that individuals with PD should be supported and helped by the community. The same findings were stated by Soilemezi et al. [17]. Most of the participants agreed that individuals with PD can lead fulfilling lives with proper treatment and support (n=306, 71.9%). These findings were in context with other studies [18], about how individuals with PD should not be stigmatized or discriminated against. 67.1% (n=186) of the participants showed agreement; these findings were in agreement with Maffoni et al. [19], and 69.5% (n=296) of the participants showed a willingness to support and help individuals with PD.

Younger ages, females, single participants, students, and occupations showed a higher level of good knowledge and awareness about PD; our findings were on the same lines as those of Pan et al. [11] and Alyamani et al. [20]. All the participants with regard to age classes showed a higher rate of information about hearing about PD, except the age group of 46-60 years old, and those above 60 years old showed inadequate information. Our findings were supported by Pan et al. [11].

Limitations of the study

Some critical gaps in knowledge were found which highlighted the need for awareness campaigns about PD. This was due to the limited population that participated in this study, more priorities are needed to increase awareness of PD, develop better infrastructure for research and management of PD, foster healthcare policy discussions for PD, and provide educational opportunities within the Kingdom, overall, we want to underline that the information presented here should only be viewed as preliminary research.

## Conclusions

The cause of PD is still unknown; however, it is likely to be a mix of biological and environmental determinants, most notably age and gender. Factors related to higher mortality in Parkinsonism may include the severity, the rate of progression, and a poor response to therapy. Some of these characteristics may contribute to the risk of a Parkinson-plus condition being misdiagnosed as idiopathic PD. Acknowledging this problem in the differential diagnosis is critical.

In general, our participants showed good knowledge about awareness of PD; however, educational efforts may be necessary to increase people's understanding of PD symptoms, treatments, and public awareness of the disease. According to the results of our study, educational programs should inform the population that PD is typically sporadic rather than familial, that non-motor symptoms are frequent in PD, and that resting tremor is a characteristic but not essential PD diagnosis.

## Appendices

**Table 7:** Questionnaire form

Section 1: Demographic Information	
Variables	Categories
Gender	Female
	Male
Age groups (years)	18-30
	31-45
	46-60
	>60
Marital Status	Single
	Married
	Divorced
	Widowed
Education Level	Primary education
	Secondary education
	Master’s degree
	Bachelor's degree
	Doctoral degree
Occupation	Student
	Unemployed
	Employed
	Retired
Section 2: Knowledge and Awareness of Parkinson's disease	
Question	Response
Have you ever heard of Parkinson's disease?	Yes
	No
Do you know what Parkinson's disease is?	Yes
	No
Which of the following is a common symptom of Parkinson's disease?	Hearing loss
	Stomach pain
	Tremors
	Vision problems
Can Parkinson's disease be cured?	Yes
	No
Which of the following is not a risk factor for developing Parkinson's disease?	Age
	Exercise
	Family history
	Smoking
Which of the following is a treatment for Parkinson's disease?	Antibiotics
	Chemotherapy
	Surgery
	Medication
Can Parkinson's disease affect a person's cognitive abilities?	Yes
	No
Is Parkinson's disease more common in men or women?	Men
	Women
	Both
What causes Parkinson's disease?	Exposure to toxins
	Genetics
	Poor diet
	All of the above
Have you or any of your family members ever been diagnosed with Parkinson's disease?	Yes
	No
Section 3: Sources of Information about Parkinson's disease	
Variables	Response
TV/Radio:	Never
	Often
	Rarely
	Sometimes
Internet:	Never
	Often
	Rarely
	Sometimes
Books/Magazines:	Never
	Often
	Rarely
	Sometimes
Healthcare professionals (e.g., doctors, nurses):	Never
	Often
	Rarely
	Sometimes
Friends/Family:	Never
	Often
	Rarely
	Sometimes
Section 4: Attitudes towards Parkinson's disease	
Variables	Response
Parkinson's disease is a result of poor lifestyle choices.	Strongly agree
	Neutral
	Agree
	Strongly disagree
	Disagree
People with Parkinson's disease should be supported and helped by the community.	Strongly agree
	Neutral
	Agree
	Strongly disagree
	Disagree
People with Parkinson's disease can lead fulfilling lives with proper treatment and support.	Strongly agree
	Neutral
	Agree
	Strongly disagree
	Disagree
More education and awareness should be provided to the public about Parkinson's disease.	Strongly agree
	Neutral
	Agree
	Strongly disagree
	Disagree
People with Parkinson's disease should be encouraged to participate in social activities and events.	Strongly agree
	Neutral
	Agree
	Strongly disagree
	Disagree
People with Parkinson's disease should be able to work and be productive members of society.	Strongly agree
	Neutral
	Agree
	Strongly disagree
	Disagree
People with Parkinson's disease should not be stigmatized or discriminated against.	Strongly agree
	Neutral
	Agree
	Strongly disagree
	Disagree
I am willing to support and help people with Parkinson's disease	Strongly agree
	Neutral
	Agree
	Strongly disagree
	Disagree
